# Local Disproportions of Quality of Life and Their Influence on the Process of Green Economy Development in Polish Voivodships in 2010–2020

**DOI:** 10.3390/ijerph19159185

**Published:** 2022-07-27

**Authors:** Grzegorz Drozdowski, Paweł Dziekański

**Affiliations:** Department of Economics and Finance, Jan Kochanowski University in Kielce, Uniwersytecka 15 Str., 25-406 Kielce, Poland; gdrozdowski@ujk.edu.pl

**Keywords:** quality of life, healthy region, healthy environment, spatial, synthetic measure, voivodship

## Abstract

Voivodships are centres of economic, social, and cultural life—they gather economic and social activities. This research aimed to evaluate the spatial differentiation of the quality of life in voivodships in Poland with the use of a synthetic measure. To achieve the research objective, the research methods used were literature analysis, statistical analysis, and synthetic measure. The Technique for Order Preference by Similarity to an Ideal Solution method was used to build synthetic measures. The choice of variables in 2010–2020 was largely conditioned by the availability of data collected in the regional system at the level of voivodships at the Local Data Bank of the Central Statistical Office. As a result of the analysis of voivodships in Poland, based on the quality of life measure, four groups were distinguished (according to the value of quartiles). In the group of the best voivodeships there were: Pomerania, Masovia, Lower Silesia, and West Pomeranian in 2010, and Masovia, Pomerania, Greater Poland, Lower Silesia, and Lesser Poland in 2020, and in the IV, the weakest group: Lodz Province, Podlasie Province, Lubusz Province, and Holy Cross in 2010, and Lodz Province, Podlasie Province, Holy Cross, and Lublin Province in 2020. The synthetic quality of life ranged from 0.37 to 0.56 in 2010 and from 0.39 to 0.64 in 2020. Regional authorities, taking care to improve economic potential, cause increasing the attractiveness of the area and attracting new entrepreneurs, create new jobs, and improve the quality of life of the inhabitants. Quality of life is shaped by economic activity and working conditions, health, education, free time and social relations, economic and physical security, and the quality of the natural environment. The results of the research conducted allow local governments to make comparisons. The conclusions drawn may allow them to identify potential directions for developing policy optimization.

## 1. Introduction

The existing connection among the economy, the existing networks of dependence and cooperation, and the increasing unification of markets has become one of the causes of the contemporary crisis [1]. It is not only a financial and economic crisis but also a social, ecological, and political crisis. The growing interconnection of regional economies creates the threat of transferring negative phenomena to related economies in the tightening cooperative network and the global system of flows (resources, factors of production, capital, goods and services, etc.) [2,3]. Development problems should be considered, inter alia, in the area of restrictions and barriers to doing business, or taking advantage of opportunities for further development.

The modern economic system is facing the challenge of responding to increasing needs while facing changes in the quality of life and climate, and the depletion of raw materials and the deterioration of our global environment [4]. A green economy allows for the harmonious management of local resources. It is an economy that contributes to improving human wellbeing and increasing social justice, while significantly reducing environmental risks and resource scarcity. It stands for a low carbon, resource sparing, and socially solid economy. A green economy means a restructuring of economic activity and infrastructure to ensure greater returns on natural, human, and economic capital. The green economy is indicated as a new model of management in which the range of ecological solutions is increased. As emphasized, this is the way to achieve sustainable development; it is not an alternative concept.

One of the tasks of local government administration units is to care for the process of sustainable development and to improve the quality of life (as the sum of a healthy region (city, commune) and a healthy environment (natural and human functioning and enterprises)), and to improve competences and social cohesion, effectively manage local resources (such as the environment, people, infrastructure, and financial resources), provide residents and economic entities with a feeling of stability, reducing the uncertainty of company operations. In the economic sphere, the priority should be building a modern industry, supporting entrepreneurship, and investing in innovations and new technologies. The indicated areas can be achieved while maintaining sustainable development consistent with environmental protection and sustainable management of resources [5].

The undeniable determinant of socioeconomic development and the quality of life is the natural environment, the resources of which constitute the foundation of economic activity and the integrity of societies. The natural environment is both the basis for the broadly understood development and is also a barrier due to the exhaustion of resources. The limitation of environmental resources in combination with the unlimited needs of people makes it necessary to manage its resources rationally. Because of this, it is possible to use the natural environment, and thus satisfy human needs, which results in obtaining an appropriate quality of life [6].

J. Piasny defines the standard of living as the entirety of the real living conditions of people, the degree of satisfaction of their material and cultural needs by a stream of goods and services, financed both from private and social funds [7]. J. Drewnowski and W. Scott define the standard of living of the population as the level of satisfaction of needs per unit of time, following as a result of goods, services, and living conditions enjoyed by the population during that unit of time [8].

Considering the issue of the quality of life, the authors decided to formulate the following research questions: What is the spatial differentiation of the quality of life in terms of regions in Poland? Does the level of quality of life depend on the level of variables characterizing the development process (endogenous socioeconomic variables)? The following questions were assessed in turn: Which endogenic potential variables shape the level of quality of life? To what extent does the financial situation shape the quality of life?

## 2. Literature Review

The European Union and numerous international organizations have included the green economy in the developed strategies. The European Union wants to create its competitive advantage in the economy, production, technological development, research and innovation by using the concept of sustainable development. The United Nations Conference on Sustainable Development RIO + 20 in June 2012 was also focused on the concept of green economy, where it was emphasized that it is necessary to redefine the economy and adopt a new model of socioeconomic development (green economy), in which special attention is given to environmental issues. Contrary to the current model (brown economy), largely based on the use of fossil fuels and other nonrenewable resources, the new model should learn from the experiences of the environmental economy and ensure the right relationship between the economy and ecosystems [9]. It is worth emphasizing that the concept of green growth is in line with the assumptions of the Europe 2020 strategy, which is based on three main pillars: smart, sustainable, and inclusive growth. Europe needs to strengthen the synergies between smart and green growth to cope with climate change, environmental and energy challenges, and increasing resource scarcity [10].

The green economy is a way of obtaining and using resources. The related structural changes in the economy are caused by the emergence of new industries of waste recycling, emission-free energy production, absorption of greenhouse gas emissions, and green urban planning. These changes should be accompanied by a parallel increase in the quality of life of the inhabitants [11]. However, a real transition to a green economy is only possible with sustainable development. The green economy includes green products and services, investments, green sectors of the economy, public procurement, and jobs. In the concept of a green economy, on the one hand, there is a certain restriction to economic processes, and on the other hand, the adjective green suggests a constant presence of ecological criteria. A new aspect of defining a green economy is the inclusion of social references and environmental aspects in every decision and production process. The concept of a green economy becomes multidimensional, pointing to the economic, social, and environmental dimensions. The green economy is more than the sum of the commitments already made: it has the potential to introduce us to a new development paradigm and a new business model where growth, development, and the environment are seen as reinforcing each other [12]. Increasing resource efficiency, promoting sustainable consumption and production, combating climate change, protecting biodiversity, combating desertification, reducing pollution, and managing natural resources and ecosystems responsibly is imperative and at the same time driving the transformation towards a green economy [13]. The indicated processes should also have a positive impact on the quality of life of the inhabitants.

The benefits of a circular green economy include better resource and ecological efficiency, a lower carbon footprint, less dependence on fossil resources, and the valorization of byproducts and waste materials from multiple sources (e.g., agroindustrial). This concept focuses on the idea of recycling, reusing, remanufacturing and maintaining a sustainable production process. A sustainable and environmentally friendly way of disposing the waste is crucial for the protection of the environment and human health. In this respect, the waste biorefinery is an example of its potential. Environmental, social, and economic problems are closely intertwined, complex and complex [14,15]. The multifaceted nature of the problems becomes a serious challenge for governments, politicians, and decision-makers [16,17]. The concept of a green economy has been criticized for significant overlap with or an attempt to replace sustainable development. Deterioration of the natural environment affects the quality of life of the population. The quality of life assessment can be positive or negative (low, medium, high quality of life; better, worse, more or less developed). The essence of nonvaluing (descriptive) interpretation consists in determining the separateness (differences) or similarities of the quality of life [18,19].

Quality of life is a complex, multifaceted category. High quality of life as the overriding goal of the concept of sustainable development should be the result of the development policy at all levels of management (national, regional, and local) [20]. Quality of life refers to the degree of satisfaction of material and nonmaterial needs of individuals and social groups, and it is defined both by objective indicators, e.g., life expectancy, the extent of poverty, the level of enrollment, and subjective indicators, e.g., the degree of satisfaction with living conditions and its various aspects, level of happiness, stress, meaning in life. The concept of quality of life is defined as multidisciplinary due to its complexity and internal connections [21]. Quality of life can be viewed in terms of human health, the state of the economy, employment, infrastructure development, crime, and the environment, both at an individual and a social level [22,23].

J. Berbeki (2005) in his works pointed to a subjective assessment of the quality of life of the inhabitants of the Małopolskie voivodship. A. Zborowski raised the issue of the level and quality of life in the space of an urban region (based on the example of Krakow). A. Sobali-Gwosdz (2004) draws attention to the size of cities, their rank and their functions in the assessment of the quality of life. M. Gotowska and A. Jakubczak (2012) emphasize the influence of local authorities on the lives of residents [24,25,26,27]. Most often, the quality of life is interpreted as the degree of satisfaction concerning material and spiritual human needs. It is shaped by many factors, the most important of which are: housing situation, employment security, health and life protection, the possibility of learning and improving qualifications, access to culture, access to commercial establishments, the condition of technical infrastructure, and the degree of satisfaction of individual needs. J. Rutkowski includes general satisfaction with life, expectations, prospects, aspirations (subjective factors), and the social and economic conditions we live in (objective factors) as factors influencing the shaping of the quality of life [28,29].

High quality of life is of particular importance in local territorial systems. How we perceive and evaluate our lives is strongly influenced by local (regional) aspects. It is the responsibility of local (regional) authorities to use the forces inherent in economic and spatial systems, i.e., social capital, together with environmental and economic potential [30,31]. Increasing the quality of life is the main goal of sustainable development. The quality of life is assessed using the housing dimension (quality of housing and housing environment) and is one of the main issues influencing the quality of life [32]. The level and quality of life of the population are influenced by numerous microeconomic factors (including human, material, and financial resources, which create a closed environment for households and the population) and macroeconomic factors (development policy, fiscal policy, unemployment, and inflation) [33,34]. Institutional factors (administration, social infrastructure and social assistance, public safety) influencing the quality of life are a relatively new area of measurement [35]. When discussing the quality of life, the most frequently analysed factors include health protection, life safety, the condition of the natural environment, the standard of living of the inhabitants, the condition of public transport and communication, the housing situation, education and training opportunities, and access to culture. An important area of the quality of life is social infrastructure and social assistance, which is the base of the social policy of the state.

Circular economy (green economy or bioeconomy—including economic activities in materials and the field of energy) shows the need for the new global society and economy to be based on renewable processes, favouring biodiversity, bringing benefits (tangible and intangible) to all people (their quality of life) now and in the future [36]. The key element is the management of limited natural resources [37]. The green economy is interpreted as ‘4R‘—i.e., reduction, reuse, recycling, and recovery. These relate to reducing resource consumption and preserving natural capital, energy resource recovery (e.g., burning waste for heating), and consumption based on continuous growth and increasing resource capacity by decoupling economic growth from environmental pressures [38,39].

## 3. Materials and Methods

This research aimed to evaluate the spatial differentiation of the quality of life in voivodships in Poland with the use of a synthetic measure. This enables the ranking and grouping of voivodeships from the point of view of the main criterion, together with examining whether and to what extent the variables of its structure determine it, and indicating the importance of local research and the information obtained as part of the research carried out at various stages of decision-making in a territorial unit. The synthetic measure (built using the Technique for Order Preference by Similarity to an Ideal Solution method) facilitates the comparison of objects in multidimensional spaces but also allows them to be organized in terms of the phenomenon under study. The factor analysis and the coefficient of variation were used to delimit the variables, and the correlation analysis was used to determine the relationships between the variables. The scatter diagrams and maps of spatial differentiation of the studied phenomenon are presented. The method of descriptive analysis was also used, together with a literature study on the quality of life and its determinants.

Empirical (measurable) data were collected in the spatial terms of Polish voivodships. The choice of variables was conditioned by the availability of secondary data collected in the Local Data Bank of the Central Statistical Office (BDL GUS) for the years 2010–2020. The research was carried out dynamically, determining the values of min {x_ij_} and max {x_ij_} for the entire research period. A voivodship should be understood as a local government unit, i.e., a regional self-government community (all inhabitants), the highest level of the basic territorial division of the country, established to perform public administration. The analysis was made on the NUTS 2 level, in Poland—voivodships.

When examining spatial differentiation in terms of the main criterion (quality of life), a certain number of diagnostic variables should be distinguished that characterize the level of the analysed phenomenon, which can be written using a multidimensional data matrix [40]. As a complex phenomenon, the quality of life was characterized based on variables illustrating, among others, social, economic, infrastructural, environmental, financial, and green economy aspects. The spatial (intraregional) polarization of the quality of life variables in the modern economy occurs primarily between cities (regions of growth in the region) and peripheral areas [41].

The selection of diagnostic variables and their verification in terms of content and/or statistics allowed for the definition of the observation matrix, which consists of objects and features. It was written as $X_{\mathrm{ij}}$:

(1) $$X_{\mathrm{ij}}=\left[\begin{array}{cccc} x_{11} & x_{12} & \ldots & x_{1m}\\ x_{21} & x_{22} & \ldots & x_{2m}\\ \ldots & \ldots & \ldots & \ldots \\ x_{n1} & x_{n2} & \ldots & x_{\mathrm{nm}} \end{array}\right],$$

where: $X_{\mathrm{ij}}$—denotes the values of the j-th variable for the i-th object, matrix of data objects, i—object number (i = 1, 2, …, n), j—variable number (j = 1, 2, …, m).

The determinants characterizing the quality of life are interactive. They are a tangle of interrelated variables that create a multidimensional space. The following variables presented in Table 1 were distinguished in the study (selected after statistical and content evaluation).

Difficulties related to the implementation of the research (selection of variables in the structure of the synthetic measure) are related, among others, to changes in legal regulations regarding the income system, the scope of tasks performed by territorial units, budget reporting, changes in the administrative division, changes in the socioeconomic situation, random events, or the lack of data collected as part of public statistics at the level of municipalities (or points, or provinces; some data were incomplete, data did not cover all voivodeships).

After determining and collecting data on the initial set of features, verification activities were undertaken according to the statistical criterion. This allowed for the elimination of quasi-constant variables. For this purpose, the coefficient of variation was used, given by the formula:

(2) $$V_{i}=\frac{S_{i}}{\bar{x}}$$

where: V_i_—coefficient of variation for the i-th variable, S_i_—standard deviation for the i-th variable, and $\bar{x}$ is the arithmetic mean of the i-th variable. Diagnostic features should show sufficient spatial variability, i.e., they should be the carrier of information differentiating the examined objects. From the set of variables, the features satisfying the inequality were eliminated $\left|V_{i}\right|\leq {\;V}^{*}$, where V* denotes the critical value of the coefficient of variation. The value V* = 0.10 was adopted as the critical value [42].

Variables strongly correlated with each other as carriers of similar information were also eliminated. It is assumed that in the case of identifying too high a value of the correlation index, a representative should be selected. The choice can be made based on the merits. The value r* = 0.75 is also taken as the threshold level of the correlation coefficient [43]. According to K. Kukuła and K. Kukuła and L. Luty, the correlation between the features does not rule out the correctness of their selection for the study, because the linear ordering of objects is based on the value of a synthetic variable. It is obtained by summing unified diagnostic variables considered important from the substantive point of view [44,45].

The selection of variables was also made based on factor analysis performed in the Statistica program (Statistica 13, TIBCO Software Inc., StatSoft Polska, Kraków, Polska). The indicated method allows for the transformation of the original set of objects into a set of their groups, using orthogonal transformations of the original data matrix (e.g., factor analysis, principal components method) [46]. It allows for a reduced number of analysed variables and transforms the old system of variables into a new system consisting of the main factors. Factor analysis is a method of studying the structure of internal relationships in multivariate observations [47,48,49].

The eigenvalues of the reduced correlation matrix, defining the variances of subsequent factors and their percentage share in the overall variability of the entire set, are presented in Table 2. After finding the presence of strong and statistically significant correlations between the analysed features, the number of factors was determined. Five factors were selected, four of them individually explain more than 10% of the total variance; the fifth explains 6.786%. Together, they explain 89.56% of the total variance.

Based on the scree criterion, the five most important factors that make up the ‘slope’ were identified as the most important. The factor loadings, similar to the coefficients included in the eigenvector, reflect the influence of individual variables on a given principal component. These are the values showing what part of the variance of a given component is constituted by the original variables.

Based on the information included in Table 2 and Table 3, it can be seen that the first main factor, exhausting 39.90614% of the total variability resource, and its eigenvalue is 9.577473. It is identified by the variables X10, X11, X18, X33, X34, X36, X38, X39, characterized by positive factor loadings.

The second factor consists of the variables X3, X4, X7, X8, X27, X29. It explains 17.48705% of the total variation resources, and the eigenvalue of this factor is 4.196891; therefore, its importance in presenting the diversity of the entire set from the mathematical point of view is the most important.

The third factor (formed by X6, X9, X25, X26, X31) accounts for 13.25567% of the common varieties of all variables, and its eigenvalue is 3.181361. The fourth factor is identified by the variables X16 (with a negative factor load), X17, X22. It exhausts 12.13364% of the common varieties of all variables, and its eigenvalue is 2.912074. The last fifth factor is characterized by negative factor loadings (variables X37, X40) exhausts 6.78696 resources of common variation, and its eigenvalue is 1.628870.

The analysis of the level of the coefficient of variation and correlation leads to the conclusion that the selected input variables are slightly correlated with other variables. The value of the coefficient of variation ranged from 0.02 (X33), −2.78 (X10) to 1.99 (X17) in 2010, and from X33 (0.02), −4.26 (X10) to 0.95 (X25) in 2020; in 2010, for the variables X17 (1.99), X25 (0.92), X26 (0.89), the lowest X3 (0.03), X33 (0.02), X10 (−2.78), and in 2020, respectively, X25 (0.95), X26 (0.84), X31 (0.74) and X33 (0.02), X17 (−0.5), X10 (−4.26). It should also be noted that the variables are positive (15 in 2010, 16 in 2020) and negative (9 and 8 variables, respectively). asymmetry W in the case of stimulants (19 variables in both extremes for the years presented) is not a favourable situation, as it means that a greater number of communes have values of these variables lower than their average.

In the next stage of the research, the direction of variable preferences regarding the general criterion under consideration was determined, dividing them into stimulants and destimulants [50,51]. Most of the variables are obvious and their determination is intuitive. In doubtful cases, it is worth using Grabiński’s procedure, which uses the fact that stimulants should be positively correlated with stimulants (the same is true for destimulants) and negatively correlated with destimulants [52]. The correctness of determining the nature of the variables can be verified by specifying the direction of correlation of individual variables with the decision variable. For a stimulant, this direction should be positive, and for a destimulant, the direction should be negative [53].

Diagnostic variables usually have different titers and different ranges of variation, which makes it impossible to compare and add them directly [54,55,56]. To make the variables comparable, the unitarization procedure was used, the purpose of which is to replace the different ranges of the variability of individual variables with a constant range [57,58].

The normalization of diagnostic variables, by the zeroed unitarization procedure, was performed depending on their types, according to the following formulas [42]:

(3) $$Z_{\mathrm{ij}}=\frac{x_{\mathrm{ij}}-\min_{i}x_{\mathrm{ij}}}{\max_{i}x_{\mathrm{ij}}-\min_{i}x_{\mathrm{ij}}},\;\mathrm{when}\;x_{i}\in S,$$

(4) $$Z_{\mathrm{ij}}=\frac{\max_{i}x_{\mathrm{ij}}-x_{\mathrm{ij}}}{\max_{i}x_{\mathrm{ij}}-\min_{i}x_{\mathrm{ij}}},\;\mathrm{gdy}\;x_{i}\in D$$

where: S—stimulant, D—destimulant; i = 1, 2 … n; j = 1, 2 … m,

maxx_ij_—maximum value of the j-th variable,
minx_ij_—minimum value of the j-th variable,
x_ij_—is the value of the j-th variable for this object,
Z_ij_—the normalized value of the jth variable for this object (belongs to the interval [0; 1]) [59,60,61,62,63].

All variables are standardized concerning the range of variability and their location in the observation space. As a result of unitarization, we obtain a matrix of feature values:

(5) $$Z_{\mathrm{ij}}=\left[\begin{array}{cccc} z_{11} & z_{12} & \ldots & z_{1m}\\ z_{21} & z_{22} & \ldots & z_{2m}\\ \ldots & \ldots & \ldots & \ldots \\ z_{n1} & z_{n2} & \ldots & z_{\mathrm{nm}} \end{array}\right],$$

where Z_ij_ is the unitary value of j-th variables for the i-th object.

A synthetic measure based on the Technique for Order Preference by Similarity to an Ideal Solution (TOPSIS) method was used to assess the spatial differentiation of the quality of life in voivodships in Poland. This measure allowed for a multidimensional and comprehensive examination at the level of the phenomenon in individual examined objects, conducting comparative analyses of objects (in spatial and time terms), and their linear ordering [64,65]. The first synthetic measure of development was proposed by Z. Hellwig to evaluate the economic development of selected countries. The synthetic measure made it possible to order the examined objects according to the level of phenomena (which cannot be measured with one measure). It provides the basis for the evaluation and comparison of multifeature objects according to the established criteria, and a comparative image between the analysed objects allows for indicating weaker and better areas of the unit’s operation. Moreover, it enables the grouping of the analysed territorial units. It can be a helpful tool for assessing the accuracy of past decisions and the effectiveness of past regional management instruments [66].

The TOPSIS linear ordering method is a reference method in which two reference points of objects in multidimensional space are determined—a pattern and an antipattern [67,68]. Their coordinates are as follows:

(a) for the pattern:

(6) $$z_{j}^{+}=\{\begin{array}{c} \max\left\{z_{\mathrm{ij}}\right\}\;\mathrm{for}\;\mathrm{stimulant}\;\mathrm{variables}\\ \min\left\{z_{\mathrm{ij}}\right\}\;\mathrm{for}\;\mathrm{destimulant}\;\mathrm{variables} \end{array}$$

(b) for the antipattern:

(7) $$z_{j}^{-}=\{\begin{array}{c} \min\left\{z_{\mathrm{ij}}\right\}\;\mathrm{for}\;\mathrm{stimulant}\;\mathrm{variables}\\ \max\left\{z_{\mathrm{ij}}\right\}\;\mathrm{for}\;\mathrm{destimulant}\;\mathrm{variables} \end{array}$$

The Euclidean distances of individual objects from the pattern and antipattern were successively calculated, according to the formulas:

(a) distances of objects from the pattern:

(8) $$d_{i}^{+}=\sqrt{\frac{1}{n}\;\overset{m}{\underset{j=1}{\sum }}{\left(z_{\mathrm{ij}}-{\;z}_{j}^{+}\right)}^{2}}$$

(b) distances of objects from the antipattern:

(9) $$d_{i}^{-}=\sqrt{\frac{1}{n}\;\overset{m}{\underset{j=1}{\sum }}{\left(z_{\mathrm{ij}}-{\;z}_{j}^{-}\right)}^{2}}$$

where: n—is the number of variables forming the pattern or antipattern, $z_{\mathrm{ij}}$—is the unitized value of the j feature for the tested unit (or the normalized value of the jth variable of the ith object), and $z_{j}^{+}/z_{j}^{-}$—is the pattern or antipattern object [69,70].

The synthetic measure for individual objects according to the TOPSIS method was determined based on the formula:

(10) $$q_{i}=\frac{d_{i}^{-}}{d_{i}^{-}+d_{i}^{+}},\;\mathrm{gdzie}\;0\leq q_{i}\leq 1,\;i=1,\;2,\;\ldots ,\;n;$$

with the proviso that: $q_{i}$ ∈ [0; 1]; $d_{i}^{-}$—is the distance of the object from the antipattern (from 0) and $d_{i}^{+}$ is the distance of the object from the pattern (from 1). A higher value of the measure indicates a better situation for an individual in the analysed area. The TOPSIS method enables the assessment to be made with the use of an unlimited number of criteria, the readability of the obtained results is high, and it allows for a linear ordering of objects (e.g., building a ranking of units in spatial terms). A significant advantage of the TOPSIS method is its computational simplicity, the indication of a positive and negative model, and a large number of alternative criteria that can be used in the evaluation process [71,72,73,74,75,76].

In the last stage of research, the division into typological groups was used to interpret the obtained measures. The first, second, and third quartiles were adopted as threshold values. The size of the synthetic measure in the first group means a better unit, and weaker units for the groups that follow [77].

The next step was to test the strength of the relationship between the variables. For this purpose, Pearson’s linear correlation coefficients were used, expressed by the formula:

(11) $$r_{\mathrm{xy}}=\frac{\sum_{i=1}^{n}(x_{i}-\overset{↼}{x})\left(y_{i}-\overset{↼}{y}\right)}{\sqrt{\sum_{i=1}^{n}{\left(x_{i}-\overset{↼}{x}\right)}^{2}\sum_{i=1}^{n}{\left(y_{i}-\overset{↼}{y}\right)}^{2}}}$$

where, r_xy_—Pearson’s linear correlation coefficient, x and y are measurable statistical features x = (1,2, … n), y = (1,2, … n), and $\overset{↼}{x},\overset{↼}{y}$ are the arithmetic average of the x and y features. The Pearson linear correlation coefficient takes the value from the interval, where, when r_xy_ = 0, there is no linear relationship, and when r_xy_ = 1 or r_xy_ = −1, there is an exact linear relationship between the features (positive or negative).

Additionally, a dendrogram is presented, a scatter plot with a fit line, and linear regression analysis and autocorrelation analysis were performed [78].

In the assessment of the synthetic measure, the cluster analysis using Ward’s minimal variance method with the Euclidean distance was also used. Its results are presented in the form of a dendrogram. This method is a hierarchical and agglomeration approach. Owing to it, it was possible to designate groups of voivodeships similar in terms of the quality of life. Hierarchical rankings of homogeneous clusters were developed for comparative purposes for the years 2010–2020. The necessary calculations were performed using the Statistica program.

To assess the impact of endogenous potentials of Polish voivodeships on the spatial differentiation of the synthetic quality of life measure, a regression model describing the dependence of the variables was estimated. Regression analysis (implemented in the Gretl program) examines the relationship between the variables of interest to us. It allows describing the relationships between the explanatory variables (Y) and the explained variables (X), between which there are more or less clear linear relationships. Linear regression analysis aims to calculate regression coefficients such that the model predicts the value of the dependent variable as well as possible so that the error of estimation is as small as possible. We describe a linear regression model by the following formula:

y_i_ = b x_i_ + a, i = 1, 2, …, n, (12)

In the case of the multiple regression model, when we have a larger number of variables, we use the following formula:

y = b_1_ x_1_ + b_2_ x_2_ +...+ b_i_ x_i_ + a, i = 1, 2, …, n, (13)

where:

b—is the regression coefficient calculated for the individual variables of the model;
x—explanatory variable;
y—is the dependent variable;
a—is an intercept.

In the process of building a regression model, high autocorrelation of variables should be excluded, and the fit of the model should be checked using the analysis of variance. Next, we move on to reading the beta standardized coefficients and their significance level. Then, we determine the percentage of the variance explained by reading the (preferably corrected) R2 statistic. The coefficient of determination determines the degree to which the estimated regression function explains the variability of the variable y. It takes values from 0 to 1. The closer to 1, the better the regression function fits the empirical data [79,80,81,82,83,84].

Spatial autocorrelation means that the values of geographically close objects are more similar to each other than distant ones. This phenomenon causes the formation of spatial clusters with similar values [85,86,87]. Positive spatial autocorrelation occurs when we observe the spatial accumulation of high or low values of the observed variables. Negative autocorrelation means neighbouring high values with low values in the space, and low values with high values. Lack of spatial autocorrelation means spatial randomness, i.e., high and low values of the observed variables are distributed independently [85]. By analysing the result of autocorrelation, it is possible to determine clusters of objects similar to each other. Knowing and understanding the structures of space enables better anticipation of changes and facilitates taking actions in development policy [88,89].

Global Moran, I statistics can be used to investigate spatial relationships [90,91]. The statistics check whether adjacent parcels form clusters with similar synthetic measure values. It was determined based on the formula [92]:

(14) $$I=\frac{\sum_{i=1}^{n}\sum_{j=1}^{n}w_{\mathrm{ij}}(x_{i}-\bar{x})(x_{j}-\bar{x})\;}{S_{o}{\;\sigma }^{2}}.$$

Local Moran statistics are negative when a given area is surrounded by regions with significantly different values of the studied variable. Positive values of the statistics should be interpreted as follows: the region is surrounded by similar regions. Owing to this, it is possible to determine clusters with low or high values of the studied variable [93,94]. The local version of the Moran statistics is the most popular analysis and is known as LISA (Local Indicators of Spatial Association). The local form of the I Moran coefficient for observations, which determines the similarity of the spatial unit to its neighbours and the statistical significance of this relationship, is determined by the formula:

(15) $$I_{i}=\frac{\left(x_{i}-\bar{x})\sum_{j=1}^{n}w_{\mathrm{ij}}(x_{j}-\bar{x}\right)}{\sigma^{2}},$$

where:

n—number of spatial objects (number of points or polygons);
x_i_, x_j_—values of the variable for the compared objects;
$\bar{x}$—average value of the variable for all objects;
w_ij_—elements of the spatial weight matrix (weights matrix standardized with rows to one),
s_o_= $\overset{n}{\underset{i=1}{\sum }}\overset{n}{\underset{j=1}{\sum }}w_{\mathrm{ij}}$,
σ^2^ = $\frac{\sum_{j=1}^{n}(x_{i}-\bar{x}){\;}^{2}\;}{n}$,—variance [95].

The Moran I statistic takes a value from the interval (−1, 1), where the value ’0‘ means no spatial autocorrelation, negative values are negative autocorrelation (<−1, 0; units with different values appear next to each other in space, differentiation of the examined objects), positive values signal a positive spatial correlation (0, >1; units with similar values occur next to each other, forming clusters) [96,97].

To illustrate the spatial dependence of the quality of life distribution in voivodships in Poland, the I Moran statistics were calculated using the Queen matrix standardized by rows to one. The calculations were made in the PQStat program.

## 4. Results

**Figure 1:**
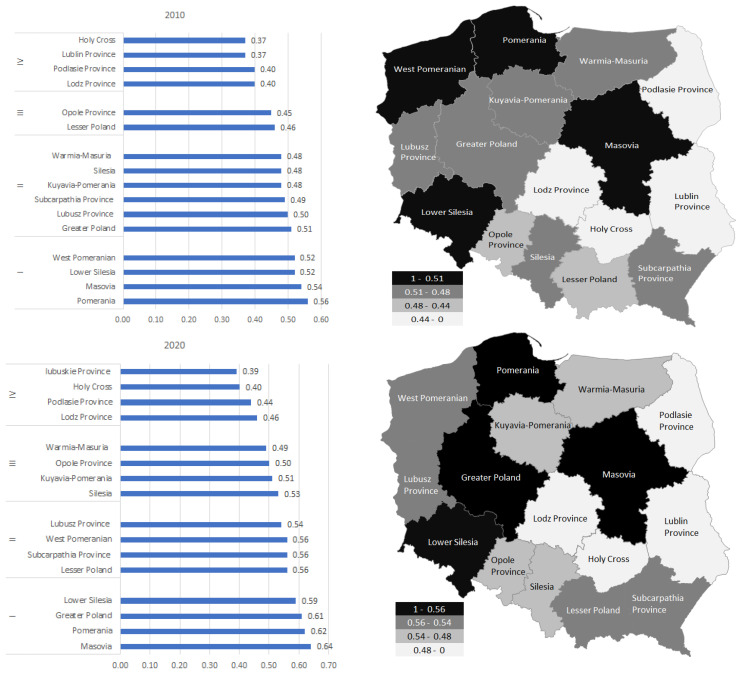

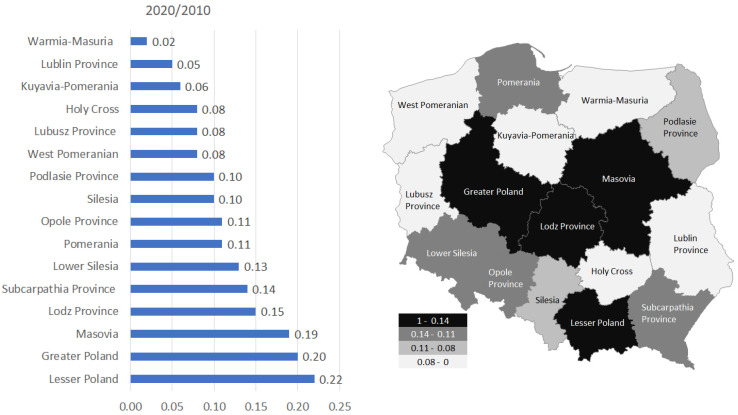
Groups and spatial differentiation of the synthetic measure of the quality of life in voivodships in Poland in 2010 and 2020. Source: own study based on the BDL CSO data.

Figure 1 presents the results of the classification of voivodeships in Poland obtained based on the synthetic measure of the quality of life. Four influences were identified, numbered, respectively, 4, 6, 2, 4 (in groups I, II, III, IV) of the voivodship in 2010 and 5, 3, 4, 4 in 2020. The best voivodships were in the group I: Pomerania, Masovia, Lower Silesia, and West Pomeranian in 2010 (Masovia, Pomerania, Greater Poland, Lower Silesia, and Lesser Poland in 2020), and in IV—the weakest: Lodz Province, Podlasie Province, Lublin Province, and Holy Cross in 2010 (Lodz Province, Podlasie Province, Holy Cross, and Lublin Province in 2020), in the light of the variables and linear ordering methods included in the study. The synthetic measure of the quality of life ranged from 0.37 (Lublin Province and Holy Cross) to 0.56 (Pomerania) in 2010 and from 0.39 (Lublin Province) to 0.64 (Masovia) in 2020. From 2020 to 2010, all voivodships recorded an increase in the value of the quality of life measure (Lesser Poland, Greater Poland, and Masovia to the highest degree). The classification of voivodships was carried out based on quartiles, which were threshold values for subsequent groups. Figure 1 shows the classification of Polish voivodeships according to the synthetic measure of the quality of life. Black indicates the group of voivodeships characterized by a better condition in the main criterion under study, the lighter the grey showing increasingly weaker units.

**Figure 2:**
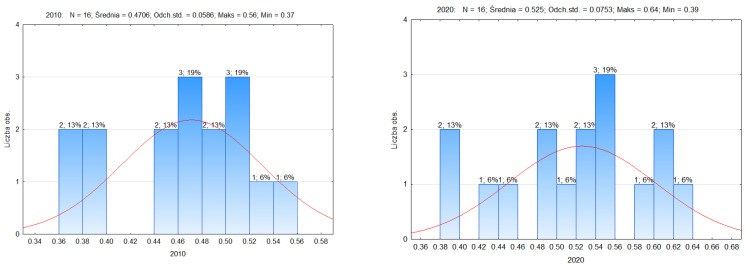
Distribution diagram showing the synthetic measure of the quality of life in voivodships in Poland in 2010 and 2020. Source: own study based on the BDL CSO data.

Figure 2 shows the number of observations and the model of the distribution of the synthetic measure of the quality of life in 2010 and 2020. The synthetic measure ranged from 0.37 to 0.56 in 2010 and from 0.39 to 0.64 in 2020. Both in 2010 and 2020 we observe lefthand skewness (As < 0). Left skew indicates that a greater number of units have values for these variables greater than their mean. The most numerous range in 2010 is 0.46–0.48 and 0.50–0.52; in both cases it was made up of three voivodeships (19%). In 2020, the most numerous range is 0.54–0.56 (three units, 19%), which means that there is a dominant feature in this range.

Statistical characteristics of the synthetic quality of life measure (Table 4) in voivodships in Poland in 2010 compared to 2020 show differentiation of the studied phenomenon. The standard deviation was 0.06 and 0.07. It indicates a slight differentiation of units in the examined aspect. The coefficient of variation (0.13–0.14) shows slight disproportions. The range (0.20–0.25) indicates the size of the dispersion between the smallest and the largest value of the variable in the studied area.

**Figure 3:**
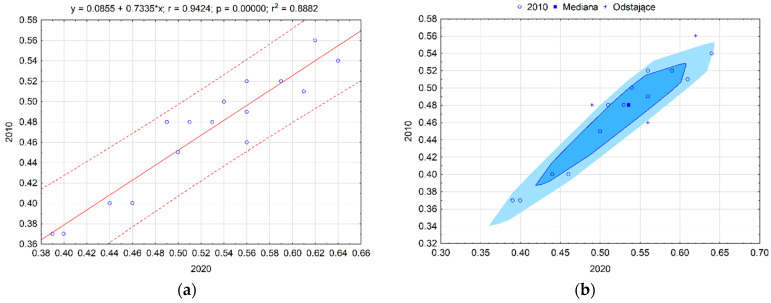
Scatter plot with the fit line (**a**) and bag chart (**b**) of the synthetic measure of the quality of life of voivodships in Poland. Source: own study based on the BDL CSO data.

Pearson’s correlation coefficient between the value of the synthetic measure in 2010 to 2020 according to the quality of life measure was 0.942. Outliers (Lublin Province and Holy Cross) were characterized by a peripheral location, agricultural function, and a weak labour market (Figure 3a). The bag chart (Figure 3b) shows statistically similar groups of the voivodship, pointing to Pomerania, Lesser Poland, and Warmia-Masuria as outliers. These are voivodships characterized by an industrial function and a good labour market.

Development is a multidimensional process that combines social, economic, cultural, political, and technical processes together with the interdependencies between them. The relations between its components are not constant and are subject to change. It is spatially diversified, which results from the location rent, i.e., the specificity of the resources, including specific natural and non-natural conditions. The location rent refers to the spatial location and endogenous territorial potential, therefore its recognition is extremely important for building social and economic cohesion [98,99,100].

The endogenous potential of an individual (including the quality of life) is built, among others, by the professional activity of inhabitants, the local labour market, entrepreneurship, infrastructure, and the condition of the natural environment. Appropriate potential contributes to an increase in the standard of living, a better social situation, and greater public safety. The level of development is not uniform. Its spatial diversity results in different conditions for running a business, or a different level and living conditions of the inhabitants [101]. Each voivodeship has its endogenous potential, which, in connection with the exogenous potential and the ability to respond to changes in the environment, may constitute an opportunity for development. Endogenous factors (specific, unique, and corresponding to a given local system [102]) are the main driving force of regional development [103,104].

The quality of life measure in the case of Polish voivodeships in the analysed years was correlated with variables in the economic, infrastructural, demographic, and natural environment. In particular, the measure of the quality of life to its structure variables was correlated with: flats equipped with water supply systems (0.7372), bathroom (0.81), network gas (0.6607), using the sewage system (0.7474) and gas network (0.6183), migration (0.7238), average monthly disposable income (0.5137), number of employees (0.7515), population per bed in general hospitals (0.5054), total waste collected during the year (0.7176), population using sewage treatment plants (0.6865), and average monthly salary (0.5363).

Quality of life is a complex process relating to many different aspects of human functioning. It is determined by many different elements, including the condition of the natural environment; access to education and culture; health and social care; safety in terms of health, loss of property, and economic terms; the quality of infrastructure; and the natural environment.

In the analysis of the green economy, elements shaping the quality of life can be found. The development of the region should be related to concern for the environment, preserving the ability of ecosystems to provide specific services and ensuring good-quality elements of the environment, which should have a positive impact on the quality of life of the inhabitants. In the area of variables describing the green economy, the quality of life measure was correlated with electricity consumption (0.2525), the share of agricultural area (−0.3907), loess (0.2767), legally protected areas (−0.2186) in the total area, waste collected selectively (0.2125) or the total annual (0.3644), mixed waste (0.6604), household waste per capita (0.6847), and the area of wild landfills (−0.2042).

An important goal of sustainable development is to improve the quality of life. Conceptually, high quality of life is the overriding goal of sustainable development [105]. The quality of life measure is also correlated with the elements shaping sustainable development, i.e., entities entered in the register (0.7921), natural persons running a business (0.727), sold production of industry (0.727), and using sewage systems (0.7474).

Another area influencing the quality of life is the financial situation. Financial resources are an essential element for the effective achievement of the unit goals in terms of current or development tasks. There is a feedback loop between the socioeconomic and financial variables [106,107]. The financial situation is the state of its finances that allows it to cover: current bills, expenses without incurring debts in a given budget period, all costs of running a business in the long term, and services at a level ensuring the safety and quality of life of residents [108]. X. Wang, L. Dennis, and Y. Sen believe that the socioeconomic environment is only one of the factors that should be taken into account when analysing the financial situation [109]. M.P. Rodríguez Bolivar and coauthors identified the main factors shaping the financial situation as the state and changes in the size of the population, the conditions of the local labour market, the needs of the local community, the size of the supply and directions of distribution of local public goods and services, and the wealth of the society, among others [110]. In the area of financial situation, the quality of life was correlated with the share of own income in total income (0.6586), the level of transfers from the state budget per capita (−0.4258), burdening own income with debt-servicing expenses (−0.3133), share in taxes constituting state budget income 0.6444, own income per capita (PC) (0.7068), income from tax of natural persons MS (0.6514), legal persons MS (0.6857), and general subsidy (−0.5289).

**Figure 4:**
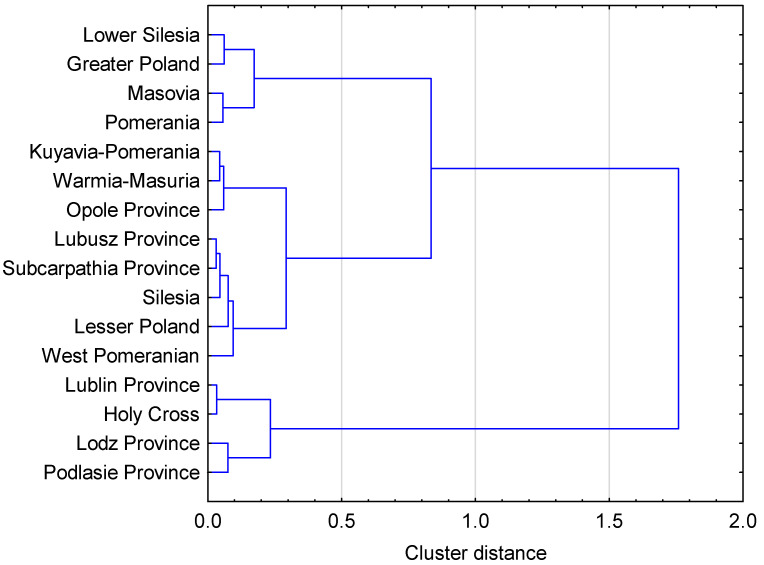
Clusters of Polish voivodships with a similar level of the synthetic measure of the quality of life. Source: own study based on the BDL CSO data.

As a result of the use of hierarchical methods, we obtain a dendrogram that illustrates the hierarchical structure of voivodships in terms of the quality of life measure. Based on the adopted features, clustering was performed using Ward’s method, taking into account the Euclidean distance between units. The result of clustering voivodeships for the analysed years, at the level of 0.5, are three groups of voivodeships most similar to each other, i.e.,

- Group I: Lower Silesia, Greater Poland, Masovia, and Pomerania (the group includes units with the highest level of synthesis and the best sizes of diagnostic plots);
- Group II: Kuyavian-Pomeranian, Warmian-Masurian, Opole Province, Lubusz, Subcarpathian, Silesian, Lesser Poland, and West Pomeranian;
- Group III: Lublin Province, Holy Cross, Lodz Province, and Podlasie Province (Figure 4).

The results of clustering may be a stimulus for further, in-depth research aimed at determining which variables had a decisive impact on the assignment of regions to individual clusters.

The impact of spatial factors on changes in the quality of life results from their location in the economic system of the region and its existing network connections. Autocorrelation makes it possible to learn about the spatial structure of the dependence of units and the interaction between the values of the studied variable in different locations. The statistics of I Moran make it possible to determine the similarities and differences between communes in general terms. By analysing the result of spatial autocorrelation, it is possible to determine clusters of objects similar to each other, but also to find objects or groups of objects that differ from their neighbourhoods.

Table 5 presents the evolution of the values of global Moran I statistics, which inform about the existence of spatial autocorrelation. A positive autocorrelation was recorded in 2010, and a slight negative spatial relationship in 2020. Based on the global values of Moran’s I obtained, it can be noticed that in the discussed period there is a decrease in autocorrelation. The decreasing value of the analysed statistics informs about the progressing process of weakening spatial dependence. This means that any observed quality of life level can appear anywhere with equal probability.

**Figure 5:**
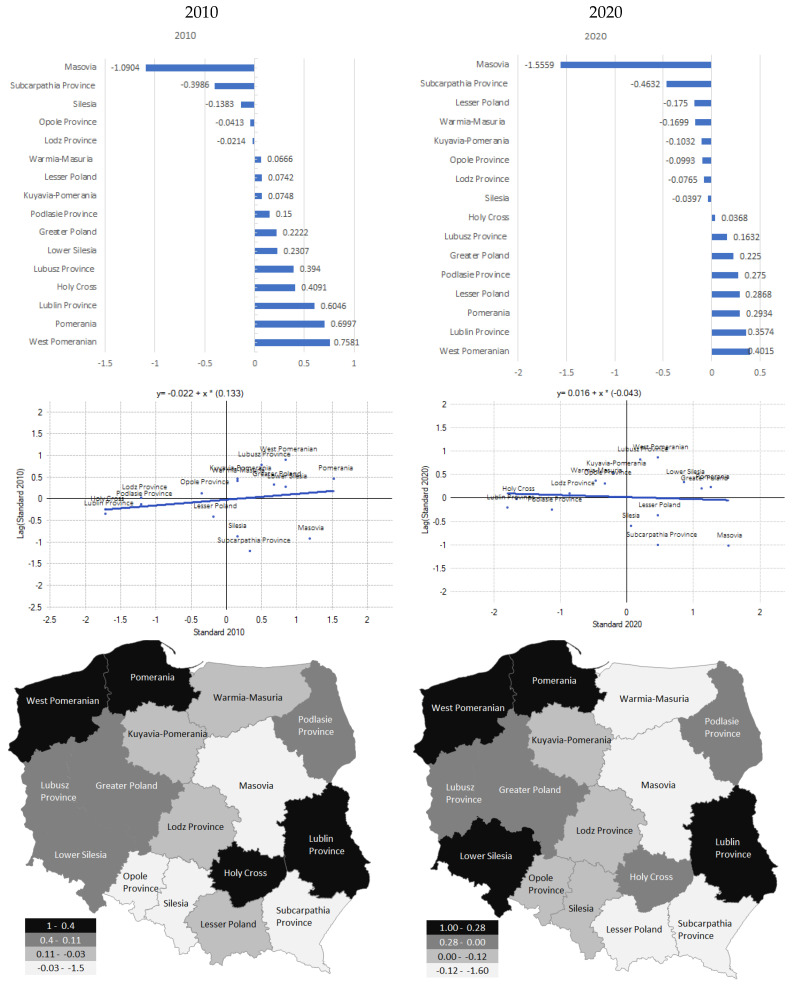
Spatial differentiation of the local value of the Moran I statistics for the synthetic measure of the quality of life in voivodships in Poland (in 2010 and 2020). Source: own study based on the BDL CSO data.

Another aspect of spatial autocorrelation analysis is the study of the shaping of variable values to neighbouring locations. Such an analysis is made possible by local statistics. The values of local statistics allow stating the existence of the so-called outliers (voivodships ’in plus‘ or ’in minus‘ from their neighbours), or clustering of objects with similar measure values. Figure 5 shows the results obtained. Significant and positive values of local Moran’s I statistics were obtained in the following voivodships: West Pomeranian (0.7581), Pomerania (0.6997), Lublin Province (0.6046), Holy Cross (0.4091), Lubusz Province (0.3940), Lower Silesia (0.2307), Greater Poland (0.2222), Podlasie Province (0.1500), Kuyavia-Pomerania (0.0748), Lesser Poland (0.0742), and Warmia-Masuria (0.0666) in 2010 (West Pomeranian 0.4015, Lublin Province 0.3574, Pomerania 0.2934, Lower Silesia 0.2868, Podlasie Province 0.2750, Greater Poland 0.2250, Lubusz Province 0.16328, and Holy Cross 0.16328 in 2020). These voivodships are surrounded by units with similar levels of potential within the studied area. Negative values of the I Moran measure were observed in the following voivodeships: Lodz Province −0.0214, Opole Province −0.0413, Silesia −0.1383, Subcarpathia Province −0.3986, and Masovia −1.0904 in 2010 (Silesia −0.0397, Lodz Province −0.0765, Opole Province −0.0993, Kuyavia-Pomerania −0.1032, Warmia-Masuria −0.1699, Lesser Poland −0.1750, Subcarpathia Province −0.4632, and Masovia −1.5559 in 2020).

To assess the impact of variables describing the quality of life area (taking into account the variables describing the green economy, waste policy, sustainable development, and financial aspect), a regression model was estimated (Table 6). The model shows the importance of the share of legally protected areas, residential areas in the total area, using the sewage system, the demographic dependency ratio for the elderly, the share of own income in total income, and personal income tax MS in the process of shaping and quality of life in voivodships in Poland. These are the variables that shape the endogenous economic base of the region. Their impact on the quality of life is varied. Their appropriate level increases the standard of living, improves the social situation, and enhances the natural environment. It can be concluded that the model fits well. (R-square determining coefficient 0.883110, corrected R-square 0.878960. The F statistic ((6, 169) 212.8002) is statistically significant (*p*-value), which means that the construction of the linear model is correct.

## 5. Discussion

Development is interpreted in terms of economic growth, but terms of demographic, social, cultural and environmental development are also important. It is to take into account one of the basic goals, which is to increase the level and quality of life, which occurs owing to positive changes in the living environment as a place to meet the needs of individuals. Systematic research on the quality of life of local and territorial communities should provide the information necessary for the authorities to evaluate and establish the regional policy. The increase or decrease in quality of life should be treated as a way of assessing the effects of local development management [111,112].

The responsibility for the conditions in which we live lies with ourselves and with the regional (local) authorities in the areas in which we live. The authorities collect funds for various reasons, and most of all for improving the quality of life. The pace of changes in the quality of life area may be decisive for some people from the point of view of migration and professional plans. The definitions of the standard of living functioning today are extremely broad and complex, which results from the combination of both measurable and nonmeasurable, objective and subjective, and quantitative and qualitative features of life, which indicates its multidimensionality (the multidimensionality of the phenomenon under study can be indicated by the number of variables in Table 1) [113].

The issues of sustainable development (or green economy) have a huge impact on all aspects of human life in economic, social, environmental, and political terms. The processes of structural transformations in economies are accompanied by an increase in the unevenness of their development [114]. Both the green economy and sustainable development are aimed at improving the quality of life by ensuring that human needs are met, and by protecting the environment, natural and social resources, and protecting the integrity of society [115].

The results obtained confirmed the spatial differentiation of the quality of life of residents and the influence of elements of the green economy on its formation. The assessment further indicates the strong influence of social and economic conditions on quality of life.

The green economy is a means to achieve sustainable development in terms of effective and purposeful use of endogenous factors of development (and activity). It helps to achieve integration between the dimensions of sustainable development (environmental, social, economic, and spatial or institutional (political)). This strengthens the context of the protection of the natural environment (conditioning socioeconomic development) and the quality of life. The green economy (which reduces carbon emissions and increases resource efficiency) must recognize state sovereignty over natural resources and must be based on resource efficiency and sustainable consumption and production patterns. The environment is the main source of resources that make life easier and foster development [116].

The sustainability (or green economy) orientation around the ’three E’s‘ (environmental protection, economic growth, and social justice) is also correlated with quality of life considerations. Quality of life refers to people’s perception of their position in life about their culture, values, and expectations. Achieving quality of life progress through sustainable development, particularly at the city level, requires careful planning that is both site- and culture-specific, and that includes contributions from communities and citizens. Improving the quality of life and meeting the needs of the present through sustainable development—introducing a green economy—should help ensure a greater probability of meeting the needs of future generations [117].

The adoption of a green economy can be useful for economic and social reasons as it has helped to reduce environmental pollution and, together with the inappropriate use of scarce resources, shapes the quality of life of the inhabitants. It benefits the region’s economy both socially and environmentally, ensuring better resource use, reducing the misuse of scarce resources, eliminating environmental pollution, and improving the region’s ecological growth [118].

The appropriate quality and structure of endogenous potential (endogenous territorial capital) ensures sustainable dynamics of regional processes and affects the level of development differentiation of individual provinces. Geographical, social, and economic conditions make the various provinces of the country characterized by a different economic situation, and thus a different level of development and quality of life.

The analysis of intraregional diversification (West Pomeranian Voivodeship) in terms of quality of life shows the need to create a system of incentives for the involvement of the private sector and the social economy in the development of social welfare, education, and social assistance—better integration and coordination of public services to increase the effectiveness of social services provided [119]. The results of quality of life research should be available to employees of city offices, city councillors, and housing estates councils, and constitute a starting point for spatial and transport planning, urban greenery and public spaces management, and revitalization activities. The diagnosis should also be available to nongovernmental organizations and socially active residents so that it can become the basis for public discussion and public participation in making decisions on budget expenditures during public consultations and submitting projects to civic budgets. In this way, systematic quality of life surveys can be an important element of knowledge-based and participatory decision-making in cities [120].

Quality of life is an interdisciplinary concept (numerous tasks and features that shape it), which makes it difficult to evaluate and define it, and indicates the need for multidimensional evaluation. The voivodship ratings obtained depend on what feature and specific quality of life symptom we take into account. However, it should be remembered that the results of the analysis depend on the diagnostic features adopted for the study (their structure and quantity) and may change when a different set is used. The selection of features is one of the tasks of particular importance because it largely determines the final results of the study, the accuracy of assessments and analyses, the accuracy of predictions, and the accuracy of decisions made on their basis. It is very difficult to collect reliable and comparable statistical data, especially in the analysis of the quality of life, which is a very complex phenomenon (also indicated by the presented studies by the authors, which indicates numerous variables characterizing the studied phenomenon) [121].

The minimum necessary condition for ecologically sustainable development is to maintain the total natural capital stock at the current level or above. The aim of ecologically sustainable development (green economy) is the wise use of natural resources in the short term so that these resources are available in the long term. Ecological sustainability cannot occur when people can deplete natural resources, leaving only polluted water and barren soil for future generations. Instead, ecological sustainability is the belief that all people must use resources wisely and efficiently, so that the resources are never exhausted or excessively polluted, which undoubtedly affects the quality of life at the current time [122,123].

Assessing the various economic, social and natural variables thus helps to explain the challenges of sustainable development (or the green economy) and highlights opportunities by linking different action plans. The indicated factors have an impact, in addition to inequality, and the development of the population, shaping the paths of economic development [124,125].

The proposed set of variables (methodology) of the quality of life analysis should make it possible to determine the quality of life measure for any country. Its use allows for quick diagnostics and identification of further trends in changes in the quality of life of residents, and comparative analysis of the quality of life in selected regions, both statically and dynamically. It should be remembered that the active cooperation of state and regional (and local) authorities with the inhabitants is needed to improve the infrastructure and create attractive living conditions [126].

## 6. Conclusions

The quality of life of the inhabitants is built, among others, by the professional activity of inhabitants, local labour market, entrepreneurship, infrastructure, and the condition of the natural environment. The appropriate potential increases the standard of living, increases production, improves the social situation, and provides greater public safety. The quality of life level is not uniform. Each voivodeship has its endogenous potential (all elements important for the economy of a given area, often of a specific and unique nature, corresponding only to a given local system), which, in conjunction with the exogenous potential and the ability to respond to changes in the environment, may constitute an opportunity for the development of a given area and quality of life.

As a result of the analysis of voivodships in Poland, based on the quality of life measure, four groups were distinguished (according to the value of quartiles). The best voivodeships were included in group I: Pomerania, Masovia, Lower Silesia, and West Pomeranian in 2010 (Masovia, Pomerania, Greater Poland, Lower Silesia, and Lesser Poland in 2020), and in group IV—the weakest: Lodz Province, Podlasie Province, Lubusz Province, and Holy Cross in 2010. (Lodz Province, Podlasie Province, Holy Cross, and Lublin Province in 2020), in the light of the variables and methods of linear ordering included in the study. The synthetic measure of the quality of life ranged from 0.37 (Lublin Province and Holy Cross) to 0.56 (Pomerania) in 2010, and from 0.39 (Lublin Province) to 0.64 (Masovia) in 2020. Quality of life as a category is complex and multifaceted; it is an interdisciplinary concept.

It is shaped by economic activity and working conditions, health, education, free time and social relations, economic and physical security, and quality of the natural environment. The epidemic state declared in Poland in 2020 as a result of the COVID-19 pandemic has affected many areas of our social and professional life. Numerous restrictions in aspects of society’s functioning have translated into a deepening of social inequalities, including perceptions of quality of life. COVID-19 also exacerbated inequalities in terms of opportunities, earned income, health care, and social security.

Systematic research on the quality of life should provide the information necessary for the authorities to evaluate and correct the manner in which social policy is conducted. The increase or decrease in synthetic measures should be treated as a way of assessing the effects of the current management of the region. The results obtained may constitute an important source of information for local government authorities on disproportions between units. The procedure described may be applied in other regions (countries). For comparison between regions, it should cover the same variables in the research areas indicated.

The results indicate the directions of new research, which include, among others, comparing the results of order based on a larger number of variables and conducting a dynamic analysis in a specific extended period to learn about the trends of changes. The results also indicate the need to analyse outliers and determine their impact on the situation of the studied area.

The added value of the article is the results of the research focused on the assessment of the quality of life of the inhabitants of voivodships in Poland in the years 2010–2020.

## Figures and Tables

**Table 1:** List of variables describing the quality of life.

	Variables	Unit	S/D *
X3	Dwellings equipped with sanitary facilities—water supply	%	s
X4	Dwellings equipped with sanitary facilities—bathroom	%	s
X6	Dwellings equipped with installations—gas mains	%	s
X7	Uses the system—water supply system	%	s
X8	Uses the system—sewage system	%	s
X9	Persons using the system—gas network	%	s
X10	Total migration balance per 1000 population	Osoba	d
X11	Total average monthly disposable income per 1 person	PLN	s
X16	Deaths per 1000 population	-	d
X17	Natural increase per 1000 population	-	s
X18	Total employed per 1000 population	Osoba	s
X22	Population per bed in general hospitals	Osoba	s
X25	Total industrial and municipal wastewater requiring treatment discharged to water or land during the year	Dam^3^/km^2^	d
X26	Industrial and municipal wastewater requiring treatment discharged to water or land during the year—treated	Dam^3^/km^2^	d
X27	Total waste collected during the year	T	d
X27	Share of the area of active landfills in the total area	%	d
X29	% Population using sewage treatment plants	%	s
X31	Share of green areas in the total area	%	s
X33	Gross enrolment rate—primary schools	-	s
X34	Average gross monthly salaries	PLN	s
X36	Own income per capita	PLN PC	s
X37	Property expenditures per capita	PLN PC	s
X38	Income from personal income tax (PIT)	PLN PC	s
X39	Income from corporate income tax (CIT)	PLN PC	s
X40	Expenditure on transport and communications PLN	PLN PC	s

For the construction of the synthetic measure, a set of 42 potential diagnostic variables collected by public statistics and related to the analysed phenomenon was originally adopted; the table shows the number of those selected for the construction of the synthetic measure as a result of the analysis. The variables were removed based on the correlation coefficient, coefficient of variation, and factor analysis; * S stimulant/D destimulant/. Source of data: a study based on the BDL CSO data.

**Table 2:** Groups of factors and their eigenvalues describe the quality of life.

Factor	Own Value	% of Total (Variance)	Cumulative (Own Value)	Cumulative (%)
1	9.577473	39.90614	9.57747	39.90614
2	4.196891	17.48705	13.77436	57.39318
3	3.181361	13.25567	16.95573	70.64885
4	2.912074	12.13364	19.86780	82.78250
5	1.628870	6.78696	21.49667	89.56946

Data source: own study based on the CSO BDL data, in the Statistica program.

**Table 3:** Values of factor loadings after rotation with the ‘Varimax’ method.

Variables	Factor (1)	Factor (2)	Factor (3)	Factor (4)	Factor (5)
X3	0.146035	**0.926340**	0.184745	0.208493	0.108775
X4	0.225769	**0.837793**	0.260945	0.372442	−0.045131
X6	0.153575	0.283894	**0.793697**	0.313021	0.217332
X7	0.017929	**0.766253**	−0.490532	−0.305696	0.136024
X8	0.101065	**0.905829**	0.191370	0.227639	−0.092560
X9	0.139483	0.216800	**0.831425**	0.305760	0.164121
X10	**0.789840**	0.265765	0.137077	0.410081	0.159171
X11	**0.795852**	0.274948	0.013686	−0.329413	−0.265372
X16	−0.048288	−0.172561	−0.198620	**−0.869399**	0.253781
X17	0.363022	0.067732	0.189999	**0.832429**	−0.146957
X18	**0.840321**	0.297020	0.251333	0.027656	0.248020
X22	−0.003097	0.396936	−0.102618	**0.775106**	0.109800
X25	0.273933	0.231362	**0.720062**	−0.410569	0.056443
X26	0.292836	0.234781	**0.724555**	−0.358166	0.089340
X27	0.348106	**0.838415**	0.037944	−0.219797	0.172228
X29	0.092625	**0.882838**	0.203478	0.143047	−0.074970
X31	−0.040598	−0.029420	**0.943407**	0.138305	0.007733
X33	**0.814294**	−0.036575	0.163372	0.017195	0.212391
X34	**0.920246**	0.080156	0.147446	−0.033572	0.160096
X36	**0.929814**	0.030116	−0.003919	0.215951	0.045051
X37	−0.296295	−0.068217	−0.070475	0.125170	**−0.904070**
X38	**0.950249**	0.230411	0.085565	0.008273	0.165767
X39	**0.937224**	0.053138	0.001038	0.151287	0.192889
X40	−0.248796	−0.008971	−0.182734	0.096070	**−0.924675**
War.wyj.	6.821100	5.182818	3.939372	3.326612	2.226768
Udział	0.284212	0.215951	0.164141	0.138609	0.092782

Extract: Principal components; Marked loads are >0.700000. Source: own study based on the CSO BDL data, in the Statistica program.

**Table 4:** Measures differentiating the synthetic measure of the quality of life in voivodships in Poland in 2010 and 2020.

	2010	2020
min	0.37	0.39
max	0.56	0.64
range	0.20	0.25
average	0.47	0.53
median	0.48	0.54
standard deviation	0.06	0.07
quartile deviation	0.04	0.04
coefficient of variation	0.13	0.14
positional coefficient of variation	0.08	0.08
quartile range	0.08	0.08
skewness (asymmetry)	−0.53	−0.41
kurtosis (measure of concentration)	−0.58	−0.59

Source: own study based on the BDL CSO data.

**Table 5:** Values of the global Moran’s I statistics for the synthetic measure of the quality of life in voivodeships in Poland (in 2010 and 2020).

Variables	2010	2020
Moran’s I	0.132925	−0.04291
Expected I	−0.06667	−0.06667
Under the assumption of normality		
Variance I	0.022125	0.022125
Z statistic	1.341839	0.159703
*p*-value	0.179648	0.873115
Assuming randomness		
Variance I	0.023059	0.023033
Z statistic	1.314393	0.156522
*p*-value	0.188714	0.875621

Source: own study based on the BDL CSO data.

**Table 6:** Results of the regression analysis of the synthetic measure of the quality of life in voivodships in Poland.

	Coefficient	Standard Error	*t*-Student’s	*p*-Value
Const.	0.289516	0.0270921	10.69	<0.0001
Share of legally protected areas in total area	0.000470627	0.000155975	3.017	0.0029
% Usage of sewage network	0.00390254	0.000260736	14.97	<0.0001
Old age dependency ratio	−0.00992326	0.000858840	−11.55	<0.0001
Residential areas in the total area	−0.0100945	0.00258376	−3.907	0.0001
Share of own income in total income	0.0762585	0.0221662	3.440	0.0007
Income tax from individuals	0.00445128	0.000461540	9.644	<0.0001
Arithmetic mean of the dependent variable	0.496080	Standard deviation of dependent variable		0.066676
Sum of squares residuals	0.090940	Residual standard error		0.023197
Coefficient of determination R-square	0.883110	Adjusted R-square		0.878960
F (6, 169)	212.8002	*p*-values for F-test		4.88 × 10^−76^
Logarithm of credibility	416.2543	Inrom. Crit. Akaike’a		−818.5086
Crit. Bayes. Schwarza	−796.3152	Crit. Hannana–Quinna		−809.5071

Observations 1–176 used; dependent variable (Y): synthetic measure quality of life. Source: own study based on the BDL CSO data.

## Data Availability

Data sharing not applicable.
